# Supplementary material for: In vivo pressure gradient heterogeneity increases flow contribution of small diameter vessels in grapevine
Source: Nat Commun. 2019 Dec 10;10:5645. doi: 10.1038/s41467-019-13673-6 (PMC6904565; doi:10.1038/s41467-019-13673-6)
Supplement: Supplementary file 1 — Supplementary Information [file 41467_2019_13673_MOESM1_ESM.pdf]

Supplementary Information for:

Bouda et al., "In-vivo pressure gradient heterogeneity increases flow contribution of small diameter vessels in grapevine"

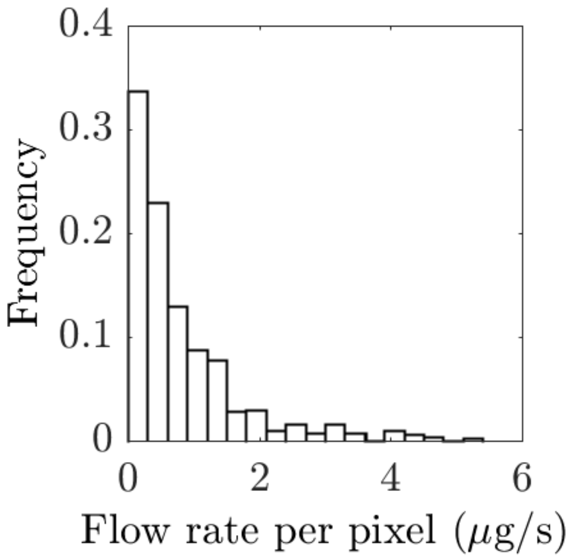

Supplementary Figure 1: Histogram of flow rates observed for MRI pixels at 78 µm resolution.

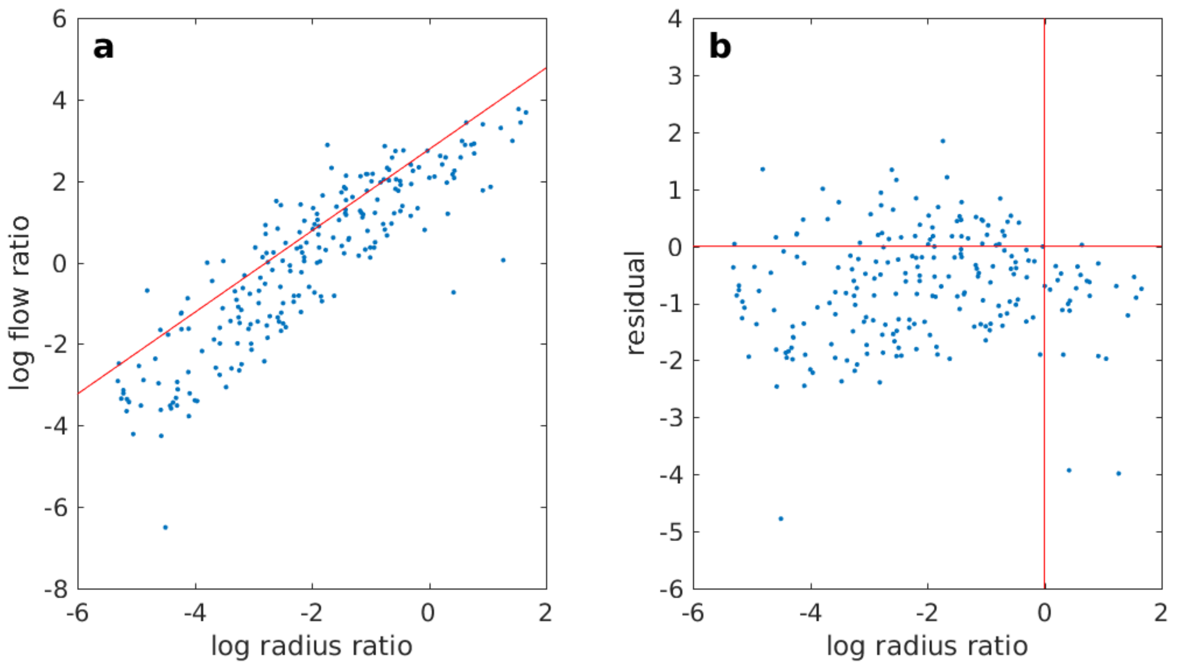

Supplementary Figure 2: Relation between volumetric flow rates and radius in vessels of the inner xylem ring. Vessel diameter and flowrate are normalized by the mean of the set. The logarithms of these unitless ratios (a) follow a line of slope 1; red line has a slope of 1 and the intercept of the bilinear surface fit to flow as a function of both radius and gradient. Residuals of individual vessels around this line (b) are roughly evenly distributed around zero for vessels below mean diameter, but virtually no residuals fall in the top right quadrant.

Supplementary Table 1: statistics (p-value, degrees of freedom, t-statistic) for separate t-tests of residuals deviating from Hagen-Poiseuille prediction, performed in moving 6 μm window along the vessel radius axis. The result is presented graphically in Fig. 7b.

Vessel radius (μm)	p value	Degrees of freedom	t-statistic
10.202	0.00060794	27	3.8797
11.202	0.00018737	36	4.162
12.202	0.0089881	39	2.75
13.202	0.0049411	47	2.95
14.202	0.00010747	47	4.2292
15.202	3.4841e-07	51	5.8529
16.202	0.0085896	48	2.7405
17.202	0.20265	49	1.2913
18.202	0.085085	56	1.7529
19.202	0.029726	54	2.2328
20.202	0.046983	55	2.0321
21.202	0.085842	57	1.748
22.202	0.8839	52	0.14674
23.202	0.9997	46	-0.0003841
24.202	0.62217	47	-0.49606
25.202	0.59361	43	-0.53762
26.202	0.73774	39	-0.33725
27.202	0.96191	36	-0.048085
28.202	0.20553	36	1.2893
29.202	0.15982	39	1.433
30.202	0.060925	33	1.9403
31.202	0.035553	37	2.1818
32.202	0.076385	33	1.8294
33.202	0.034872	29	2.2137
34.202	0.03694	26	2.1994
35.202	0.0096395	20	2.8619
36.202	0.0049629	17	3.2259
37.202	0.0050452	11	3.4915
38.202	0.00018986	11	5.4872
39.202	0.00017616	10	5.7865
40.202	1.3814e-05	12	7.0269
41.202	1.3031e-05	12	7.0687
42.202	4.7564e-06	13	7.4613

Vessel radius (μm)	p value	Degrees of freedom	t-statistic
43.202	3.2772e-06	13	7.724
44.202	6.9572e-07	13	8.8857
45.202	2.2341e-05	10	7.4308
46.202	0.0018111	8	4.5761
47.202	0.0034437	8	4.0988
48.202	0.0050042	5	4.7724
49.202	0.011843	4	4.3835
50.202	0.010662	4	4.5195
51.202	0.010662	4	4.5195
52.202	0.0058061	3	7.0751
53.202	0.035246	2	5.1847
54.202	0.0014563	4	7.8018
55.202	0.0014563	4	7.8018
56.202	0.0014912	3	11.288
57.202	0.0014912	3	11.288
58.202	0.013361	2	8.5644
