# Supplementary material for: In vivo pressure gradient heterogeneity increases flow contribution of small diameter vessels in grapevine
Source: Nat Commun. 2019 Dec 10;10:5645. doi: 10.1038/s41467-019-13673-6 (PMC6904565; doi:10.1038/s41467-019-13673-6)
Supplement: Supplementary file 3 — Reporting Summary [file 41467_2019_13673_MOESM3_ESM.pdf]

nature research

Corresponding author(s): Martin Bouda

Last updated by author(s): Nov 12, 2019

Reporting Summary

Nature Research wishes to improve the reproducibility of the work that we publish. This form provides structure for consistency and transparency in reporting. For further information on Nature Research policies, see Authors & Referees and the Editorial Policy Checklist .

Statistics

For all statistical analyses, confirm that the following items are present in the figure legend, table legend, main text, or Methods section.

n/a	Confirmed	
☐	☒	The exact sample size (*n*) for each experimental group/condition, given as a discrete number and unit of measurement
☐	☒	A statement on whether measurements were taken from distinct samples or whether the same sample was measured repeatedly
☐	☒	The statistical test(s) used AND whether they are one- or two-sided<br>*Only common tests should be described solely by name; describe more complex techniques in the Methods section.*
☐	☒	A description of all covariates tested
☐	☒	A description of any assumptions or corrections, such as tests of normality and adjustment for multiple comparisons
☐	☒	A full description of the statistical parameters including central tendency (e.g. means) or other basic estimates (e.g. regression coefficient) AND variation (e.g. standard deviation) or associated estimates of uncertainty (e.g. confidence intervals)
☐	☒	For null hypothesis testing, the test statistic (e.g. *F*, *t*, *r*) with confidence intervals, effect sizes, degrees of freedom and *P* value noted<br>*Give P values as exact values whenever suitable.*
☒	☐	For Bayesian analysis, information on the choice of priors and Markov chain Monte Carlo settings
☒	☐	For hierarchical and complex designs, identification of the appropriate level for tests and full reporting of outcomes
☒	☐	Estimates of effect sizes (e.g. Cohen's *d*, Pearson's *r*), indicating how they were calculated

Our web collection on statistics for biologists contains articles on many of the points above.

Software and code

Policy information about availability of computer code

Data collection	Raw MicroCT X-Ray data were reconstructed using Octopus (v8.3) software (Institute for Nuclear Sciences, University of Ghent, Belgium) and processed into a vessel network graph using Avizo (v6.2) software (VSG Inc., Burlington, MA, USA), before network analysis for flow simulations by the custom Tomography-derived Automated Network Analysis of Xylem (TANAX) software. These steps and the custom code are fully described by Brodersen et al. (2011) New Phyt. 191:1168-1179. TANAX source code is available at https://bitbucket.org/xylemlab/tanax). NMR data were collected and processed to the baseline images for the analysis using IDL (v8.5.1) software (Exelis Visual Information Solutions, Inc., Boulder CO.).
Data analysis	All analysis was performed with novel custom code using MATLAB (R2017b, MathWorks, Inc., Natick, MA) and a FORTRAN routine for simulating flows, which are both made available and described separately in the Code availability statement in the manuscript. It is deposited at a public repository described there.

For manuscripts utilizing custom algorithms or software that are central to the research but not yet described in published literature, software must be made available to editors/reviewers. We strongly encourage code deposition in a community repository (e.g. GitHub). See the Nature Research guidelines for submitting code & software for further information.

Data

Policy information about availability of data

All manuscripts must include a data availability statement. This statement should provide the following information, where applicable:

- Accession codes, unique identifiers, or web links for publicly available datasets
- A list of figures that have associated raw data
- A description of any restrictions on data availability

All calculation inputs (including raw MRI images) and outputs, including all data reported in figures here are available online at https://bitbucket.org/xylemlab/invivoflow/. The reconstructed 3D microCTµCT scan is available at Open Science Framework repository with DOI 10.17605/OSF.IO/EY2TJ [https://osf.io/ey2tj/?view_only=17a39b03d05b4562a98a05ec70caca04].

Field-specific reporting

Please select the one below that is the best fit for your research. If you are not sure, read the appropriate sections before making your selection.

☐ Life sciences ☐ Behavioural & social sciences ☒ Ecological, evolutionary & environmental sciences

For a reference copy of the document with all sections, see nature.com/documents/nr-reporting-summary-flat.pdf

Ecological, evolutionary & environmental sciences study design

All studies must disclose on these points even when the disclosure is negative.

Study description	We first visualized the flow of water through individual vessels within the xylem network of an intact grapevine using NMR imaging. We then excised the piece stem segment that was visualized with NMR and imaged with high resolution X-ray micro-computed tomography to digitally extract and study the connectivity of the vessel network. We then used the NMR images of real flows through the network to predict the pressure gradients that would be required to produce the same flow rates simulated from the microCT-derived network.
Research sample	We chose grapevine because it is a model species within the field of plant physiology for understanding the hydraulic conductivity of plants. It is also amenable to working with the NMR instrument, which requires plants with long, narrow diameter stems that are able to be placed into the bore of the magnet. There are few species where this type of experiment are possible.<br><br>A population of plants from which we selected the plant for the study were grown in a greenhouse from commercially available vineyard stock.<br><br>The study was conducted on a single (one) grapevine (Vitis vinifera L. , 'Cabernet Sauvignon' scion grafted on to SO4 rootstock) plant approximately 2.5m in length. Because of the technical difficulty, expense, and limited access to both the NMR and microCT experiments, we chose to work with a single sample. Within this stem, we visualized all (n=491) vessels of the xylem network in the scanned stem section, allowing for a full description of the vessel network following the null hypothesis (flow rates in individual vessels follow Hagen-Poiseuille flow thanks to transverse consistency in the pressure gradient) and thus a conclusive test of it for the given stem. Thanks to the vessel census, the study design was sufficient to demonstrate that vessel radius and overall axial gradient are not sufficient for accurate prediction of of in vivo flow rates in each vessel.<br><br>While our limited sampling design at stem scale does not allow us to demonstrate that xylem is in general heterogeneous, it does allow us to demonstrate that it is not in general homogeneous. The manuscript discussion further provides strong theoretical reasons underpinning our belief that multiple stem replicates would yield similar results, despite differences between plants based on specific xylem network properties. Due to the limited sampling design here, the range of variation in the heterogeneity in the population of grapevine, let alone angiosperm, xylem requires further quantification. A general assumption of homogeneity, however, can be excluded on the basis of present data alone.
Sampling strategy	As noted above, the sampling strategy at vessel scale was a census while sample size at stem scale was predetermined by the technical, logistical, and financial constraints involved in the technology used, which allowed us to perform the experiment only on a single plant. The latter does constrain the ability to generalise our results, but is nevertheless sufficient, based on the experimental design and hypothesis formulation, to exclude the null hypothesis.
Data collection	Carel Windt performed the NMR imaging experiment. Craig Brodersen and Andrew McElrone performed the microCT imaging and initial image reconstructions, and developed the xylem network parameter extraction software. Martin Bouda developed the image processing routines specific for this project, the modeling framework and integration into MatLab, and the related simulations. All authors contributed to the development of the hypotheses, experimental design, modeling approach, data analysis, and manuscript preparation.
Timing and spatial scale	NMR and microCT imaging were performed in 2014. These datasets were then used for model development, implementation, and data analysis between 2016 and 2018.
Data exclusions	No data were excluded.
Reproducibility	No attempts to repeat the experiment were made. A full description is provided to allow reproducibility.
Randomization	Because n=1 stems and n=N (all 491) vessels, traditional statistical procedures are not applicable. For testing for differences in network properties between xylem domains, vessels were allocated into groups (dorsal/ventral or lateral, inner or outer) based on a previous understanding of grapevine xylem structure (Brodersen et al., 2013, AJB 100(2):314-321), observed disconnection and distance across the inner/outer divide and maximising the differences in mean radius between dorsal/ventral and lateral zones under the constraint of radial symmetry. The main result (deviation from simple HP flow) is unaffected by these divisions, however, as it holds both on the entire xylem and the groups considered separately.
Blinding	Blinding was not relevant to our study as all data handling and analysis leading up to and including hypothesis testing was performed by software.

Did the study involve field work? ☐ Yes ☒ No

Reporting for specific materials, systems and methods

We require information from authors about some types of materials, experimental systems and methods used in many studies. Here, indicate whether each material, system or method listed is relevant to your study. If you are not sure if a list item applies to your research, read the appropriate section before selecting a response.

Materials & experimental systems

n/a	Involved in the study
☒	☐ Antibodies
☒	☐ Eukaryotic cell lines
☒	☐ Palaeontology
☒	☐ Animals and other organisms
☒	☐ Human research participants
☒	☐ Clinical data

Methods

n/a	Involved in the study
☒	☐ ChIP-seq
☒	☐ Flow cytometry
☒	☐ MRI-based neuroimaging
